# Draft Genome Sequence of the Emerging Bivalve Pathogen *Vibrio tubiashii* subsp. *europaeus*

**DOI:** 10.1128/genomeA.00625-16

**Published:** 2016-07-28

**Authors:** Edward J. Spinard, Javier Dubert, David R. Nelson, Marta Gomez-Chiarri, Juan L. Barja

**Affiliations:** a Department of Cell and Molecular Biology, University of Rhode Island, Kingston, Rhode Island, USA; b Departamento de Microbiología y Parasitología, CIBUS, Facultad de Biología, Universidade de Santiago de Compostela, Santiago de Compostela, Spain; c Department of Fisheries, Animal and Veterinary Sciences, University of Rhode Island, Kingston, Rhode Island, USA

## Abstract

*Vibrio tubiashii* subsp. *europaeus* is a bivalve pathogen isolated during episodes of mortality affecting larval cultures in different shellfish hatcheries. Here, we announce the draft genome sequence of the type strain PP-638 and describe potential virulence factors, which may provide insight into the mechanism of pathogenicity.

## GENOME ANNOUNCEMENT

*Vibrio tubiashii* subsp. *europaeus* is an emerging bivalve pathogen identified recently as the etiological agent responsible for larval and spat mortalities in clam, oyster, and abalone cultures detected in Spanish and French hatcheries (1, 2). This pathogen is a causative agent of vibriosis, inducing mass mortalities and important economic losses, representing the main bottleneck for the production process in shellfish aquaculture (1, 2).

*V. tubiashii* subsp. *europaeus* PP-638^T^ (= CECT 8136^T^ = DSM 27349^T^) was originally isolated from a culture tank of flat oyster (*Ostrea edulis*) during an episode of larval mortality in a shellfish hatchery (Galicia, Northwest Spain) (1). DNA was isolated from *V. tubiashii* subsp. *europaeus* PP-638^T^ grown overnight in YP30 using the Wizard genomic DNA purification kit (Promega), according the manufacturer’s instructions, except DNA was resuspended in 2 mM Tris-HCl buffer (Bio Basic). Genomic DNA was sequenced using an Illumina MiSeq at the Genomics and Sequencing Center at the University of Rhode Island, Kingston, RI. Reads were trimmed using CLC Genomics Workbench (version 8.5.1) for quality, ambiguous nucleotides, and adapters. A total of 2,943,708 paired-end and 3,234,516 mate-paired reads providing 199× coverage were assembled using SPAdes (version 3.1.1), using default parameters (3). Contigs were filtered based on 34× coverage and 4,000-bp length, resulting in 10 contigs with an *N*_50_ of 1,788,614 and an average G+C content of 45.37%. The assembly was mapped to *Vibrio tubiashii* ATCC 19109 using the CLC Microbial Genome Finishing module and resulted in six contigs mapping to chromosome 1, one complete contig representing chromosome 2, one complete contig representing the p251-like megaplasmid, and one contig mapping to the p57-like plasmid (4). One 4,885-bp contig did not map to the reference genome. The draft genome was submitted to Rapid Annotations using Subsystems Technology (RAST) for annotation, resulting in 5,157 open reading frames (5–7).

Encoded on chromosome 2 of the *V. tubiashii* subsp. *europaeus* PP-638^T^ genome is a putative metalloprotease with 75% similarity to VtpA found in *Vibrio coralliilyticus* RE22 (8). Another protease with 71% similarity to Epp in *Vibrio anguillarum* M93Sm is encoded on chromosome 2 (9). Three putative hemolysins and phospholipases are encoded in the genome. One hemolysin located on chromosome 2 has 67% similarity to Plp in *V. anguillarum* M93Sm (10, 11). In *V. anguillarum* M93Sm, *plp* is divergently transcribed from the pore-forming hemolysin/cytolysin *vah1* (11). In *V. tubiashii* subsp. *europaeus* PP-638^T^, the Plp homolog is also divergently transcribed away from a pore-forming cytolysin, although it has 42% similarity to aerolysin in *Aeromonas eucrenophila* (NCBI Reference Sequence WP_042642875.1), not *vah1*. The genome encodes two secretion systems (type III secretion system [T3SS] and T6SS) that are used to deliver effector molecules directly into the host. The T3SS-secreted virulence factor has a domain similar to the GTPase-activating domain found on YopE from *Yersinia pestis* (12–16). While the T6SS structural components are encoded on the p251-like megaplasmid, the protein responsible for forming the puncturing tip of the T6SS secretion system, VgrG, appears to be encoded by two genes. One VgrG-encoding gene is on chromosome 1, and the second is on chromosome 2.

### Nucleotide sequence accession numbers.

This whole-genome shotgun project has been deposited in DDBJ/EMBL/GenBank under the accession no. LUAX00000000. The version described in this paper is the first version LUAX01000000.
